# The influence of the language style of the anchor on consumers’ purchase intention

**DOI:** 10.3389/fpsyg.2024.1370712

**Published:** 2024-06-21

**Authors:** Zhen Li, Suqin Zheng

**Affiliations:** Business School, Putian University, Putian, Fujian, China

**Keywords:** language style, appeal to emotion, appeal to logic, practical product, hedonic product

## Abstract

Remarkably, e-commerce anchors have become one of the hot careers in the new media era. As a link between goods and consumers, anchors affect the willingness of consumers to purchase, which eventually impacts the sales volume of commodities in the live broadcast. Therefore, the language style of anchors is of vital significance. However, local and foreign research rarely investigates the interaction between the language style of anchors and different product types and the influential mechanism on consumers’ purchase willingness. In light of the SOR theory’s logic and from the viewpoint of consumer perceived value, this research study scrutinizes the interaction between the language styles of different authors (appealing to emotion and appealing to logic) and different types of products (hedonic products and practical products), as well as the effect mechanism on the consumers’ willingness to purchase. Using questionnaire surveys and empirical analysis, this paper intends to analyze the inherent correlation between study variables, in order to extend valuable suggestions for enterprise practice.

## 1 Introduction

Based on the leadership style framework proposed by Bass (1967), Sheth (1976) introduced the notion of communication style; thereby, dividing the interaction between purchasers and sellers into the content of communication and style of communication. Content of communication refers to the key content articulated by persons, while style of communication refers to how both parties express themselves during interactions (Jing et al., 2020). The influence of language style on communication effectiveness has received successive attention in linguistics, psychology and communication studies, Studies on communication style involve various types of research, including goal-oriented communication style analysis (Sheth, 1976), such as task-Oriented Style, Interaction-Oriented Style, Self-Oriented Style, communicator image-based communication style analysis (Norton and Pettegrew, 1977), such as dominant, open, dramatic, relaxed, contentious, animated, friendly, attentive, impression-leaving; power difference-based communication style analysis (Erickson et al., 1978), such as powerful style and powerless style; expression differences -based communication style analysis, such as Literal and Figurative (Kronrod and Danziger, 2013), abstraction and specification (Massara et al., 2020), similes and metaphors (Chang and Yen, 2013), Cold and warm (Xianzheng et al., 2023). In the field of marketing, studies related to language style include the following two aspects: First, studies related to advertising language style. Massara et al. (2020) found that abstract language style advertisements are more suitable for lifestyle-based advertising content, while concrete language style advertisements are more suitable for product-based advertising content (Massara et al., 2020). Chang and Yen (2013) found that metaphorical language style advertisements are more suitable for hedonic products, and explicit language style ads are more suitable for practical products. Second, related studies on online review language styles. Zhenzhong et al. (2023) found that for consumers with task motivation tendency, online reviews with declarative language style enhance consumers’ purchase intention through factual perception, and for consumers with entertainment motivation tendency, online reviews with rhetorical language style enhance consumers’ purchase intention through fun perception. Consumers’ shopping motivational tendencies have moderating effects. Aerts et al. (2017) concluded that online reviews with concrete language styles are more trustworthy and influential compared to online reviews with abstract language styles. Liu et al. (2018) found that compared to online reviews with objective language styles, online reviews with subjective language styles have a significant effect on purchase intentions of men in hedonistic contexts and female purchase intention in a utilitarian context had a positive influence.

In the context of social media, live broadcasting has gradually become an important channel for brand promotion, commodity sales, and the establishment of connections between enterprises and customers, and the anchor is the organizer of the live broadcast, the presenter of the content, and the guide of the transaction (Wang Cuicui et al., 2023). From the customer’s point of view, the anchor is an enterprise shopping guide who shows product details, explains product attributes, and conveys the experience of use; from the enterprise’s point of view, the anchor is an enterprise salesperson who conveys brand concepts and values, plans and executes live broadcasting programs, and guides customers to shop and place orders. Compared with the spokesman, the anchor has the following three advantages: First, more interactive. Live consumption scenarios brand anchor and live in the audience can two-way, diversified, real-time interaction, live in the audience through the pop-up or voice to express the needs of the brand anchor through the language, product display to answer questions; Second, the social sense of presence is stronger. Live consumption scenarios brand anchors can realize the full range of product display through product introduction, product trial, experience sharing (i.e., cognitive social presence), but also through the product and brand story, social hotspots and topics, user pop-up interactions and exchanges to pull into the distance between the customer (i.e., emotional social presence) (Yanping et al., 2023); Third, more flexible. Compared with traditional advertising and online comments can only present a specific, static, single language style, the language style of the anchor in the live room can be more flexible, the same anchor that can change the language style according to the audience characteristics, but also through the replacement of the anchor, the anchor and the vice-anchor mixed and matched with the other live room with the other live room, invite the stars to enter the live room and other diversified ways to realize the flexibility and diversity of the language style. Therefore, there are three major shortcomings in the existing research: Firstly, live broadcasting scenarios are highly interactive, strong sense of social presence and flexibility so that the language style of anchors shows greater influence, so what types of language style of anchors are included, and what are the links and differences between them and the language style of advertisements and commentaries that are worth further research; Secondly, furthermore, is there any difference in the effect of different language styles on different types of products (practical products or hedonic products)? What are the mechanisms that influence consumer purchases? Third, furthermore, are there differences in the effects of different language styles on different user groups (independent egos or interdependent egos), and how should anchors adjust their language styles to suit the preferences of live viewers? The above questions remain to be addressed.

In the context of consumer perceived value, this research article employs the SOR theory as the logical foundation, in order to investigate the interaction between diverse styles of host language (appealing to emotion and appealing to logic) and different types of products (hedonic products and practical products), and investigate the interaction between diverse styles of host language (appealing to emotion and appealing to logic) and Different population characteristics (independent self or interdependent self),as well as the influential mechanism on the purchase intentions of consumers. Using questionnaire surveys to gather data and perform empirical analysis, this paper attempts to explore the inherent correlation between different understudied variables. Thus, there are multiple key innovations associated with this research article: firstly, the language style of the host in the live broadcast scenario is summarized into 2 major types including appealing to logic and appealing to emotion, eventually, simplifying the present complex and diverse classification approaches. Secondly, this study empirically tests the mechanism of the host language style by establishing that the host language style exerts a positive effect on the purchase willingness of consumers through consumer perceived value. Meanwhile, consumer perceived value plays a significant mediating role in this process. Thirdly, this paper reports that the different types of products (practical products and hedonic products) and Different population characteristics (independent self or interdependent self) play a moderating role in the association between host language style and consumer perceived value; hence, expanding the theoretical boundary of language style-related studies.

## 2 Literature review

### 2.1 Classic theories of communication styles

#### 2.1.1 Communication style based on goal orientation

Sheth (1976) advocated that the interaction between sellers and purchasers is a function of the style and content of the communication. In line with Bass (1967) framework of leadership style, Sheth (1976) put forward the concept of communication style, which refers to the habits, forms, and rituals adopted by consumers and vendors in their interactions, including task-oriented style, self-oriented style, and interaction-oriented style. On the one hand, task-oriented style refers to the individuals who are most interested in the efficiency of the task at hand, intending to minimize time, costs, and workload. On the other hand, the interaction-oriented style posits that social interaction serves as an integral part of the interaction process, and values establishing a personal affiliation with others. In the meantime, the self-oriented style reflects the person’s focus on themselves in interactive situations, being more concerned regarding their own contentment and displaying less empathy for others (Sheth, 1976). Accordingly, Xinhua and Yaoqi (2017) highlighted that people with interaction-oriented communication styles discuss common hobbies and interests with each other; thereby, offering praise and recognition to each other. Although, task-oriented customers are highly goal-oriented and determined in terms of their communication with personnel (Xinhua and Yaoqi, 2017). Keeling et al. (2010) found that Avatar interaction programming avoids negative evaluations of salespeople due to self-directed styles, and also found that Avatar interaction programming using either social or task-oriented communication styles also found that Avatar interaction programming using either social or task-oriented communication styles can help to enhance user trust and realize patronage intentions.

#### 2.1.2 Communication style based on the image of the communicator

Evidently, Norton and Pettegrew (1977) defined communicator style as an individual’s way of indicating how to comprehend, interpret, filter, or realize the literal meaning of words through oral or verbal interactions. Operationally, the communicator style was described based on the 9 explanatory/independent variables, which were open, relaxed, dramatic, animated, attentive, friendly, dominant, contentious, and impression-leaving. Consistent with this, the communicator image was selected as the explained variable (Norton and Pettegrew, 1977). Furthermore, Notarantonio and Cohen (1990) advocated that the more open the salesperson, the higher the level of self-disclosure of the salesperson as a communicator, and the more likely the conversation focus is on the salesperson themselves. As a consequence, customers are more difficult to make purchasing decisions due to insufficient product information. In contrast, subjects in a high-dominant position were more inclined to purchase products, as compared to the customers in a low-dominant position, since high-dominant salesperson occupied a dominant position in communication; thus, facilitating the dissemination of sufficient product-oriented information for customers to make buying decisions (Notarantonio and Cohen, 1990).

#### 2.1.3 Communication style based on the rights differences

In specific, speaking style is associated with variables such as the speaker’s identity (Fischer, 1958), social class (Labov, 1972), etc. Reportedly, Erickson et al. (1978) confirmed that persons with low social status and power frequently tend to utilize emphatic language (“very,” “definitely,” such as “I definitely did it”), gestures (such as using hands and expressions such as “over there” when speaking), hedging (“a little,” “I think,” “I guess,” etc.), especially formal grammar, hesitant forms (“um,” “you know,” etc.), questioning forms (including using interrogative and rising tones in declarative contexts), and polite forms (“thank you,” “please,” etc.), known as the “powerless” style of testimony. Individuals with comparatively high social power in the courtroom (such as doctors, parole officers, and other professionals) not only rarely employ these “powerless” features, but also speak in a more direct manner, which Erickson et al. (1978) refer to as the “powerful” style. Similarly, language forms that stimulate power judgments are known as dominant and subordinate languages. Noticeably, Johnson (1987) defines dominant and subordinate languages based on the impressions made by these languages on the listener. Moreover, speech characterized by hedging, directive phrases, emphatic words, and hesitant forms (“ah,” “er,” etc.) represents powerless language, whereas powerful language does not constitute such forms (Erickson et al., 1978).

### 2.2 Communication style based on rhetoric theory

Based on Aristotle’s rhetoric, Wei et al. (2016) categorized language styles into 5 major types, combined with the grounded theory: appeals to credibility, appeals to emotion, appeals to logic, appeals to exaggeration, and appeals to reward. Primarily, appeals to credibility illuminate the authority of the information’s source; further, appeals to emotion add emotional information to persuasive messages; additionally, appeals to logic refer to the speaker’s use of logical reasoning to reach conclusions; besides, appeals to exaggeration most often utilize exaggerated or even boastful descriptions; finally, appeals to reward typically promise to reward with virtual or physical goods (Wei et al., 2016). Toto et al. (2020) put forward Aristotle’s rhetorical theory as a research framework, in order to propose 6 different types of language styles from three different aspects, namely: appeals to logic, appeals to personality, and appeals to emotion. The aforementioned aspects include credibility, promise, assertiveness, argument structure, argument clarity, and reverse incentive. Particularly, credibility is manifested in the persuaders’ capability to convince the persuaded that persuaders carry a specific stance on a certain issue based on their own experience. In certain, assertiveness is manifested in the persuader’s often dominant position, issuing orders to influence the attitudes and behaviors of the audience. Simultaneously, credibility and assertiveness are consistent with the notions of appeals to personality. On the one hand, argument structure refers to the logical structural connection between information; while, on the other hand, argument clarity refers to the logical clarity of information. At the same time, argument structure and argument clarity are consistent with the concept of appeals to logic. Besides this, a promise is directly manifested as extending welfare rewards to the audience, in order to arouse positive emotions; conversely, a reverse incentive is executed by creating fear to arouse the audience’s nervousness and trigger the urgency of demand. Concurrently, promise and reverse incentives are aligned with the notion of appeals to emotion (Toto et al., 2020). Aligned with Hovland’s persuasion model and grounded theory, Gang and Qiansha (2022) categorized the language persuasion styles of anchors into 5 different categories, namely: appeals to emotion, appeals to personality, appeals to logic, appeals to reward, and appeals to exaggeration, and depicted the effect of the anchors’ language characteristics in the social e-commerce environment on product sales. The derived results illuminated that the language style of appeals to personality demonstrated the strongest positive effect on the product’s sales volume; further, the language style of appeals to emotion exhibited a significant positive influence on the product’s sales volume; and lastly, the language style of appeals to logic displayed a negative impact on the product’s sales volume. In addition to this, the study outcome indicated that the same language style recorded diverse effects on different types of products (Gang and Qiansha, 2022).

Different scholars have different views on communication style, but there are overlaps and similarities between the conceptual connotations. For example, ‘resorting to credibility ‘specifically enhances the credibility of information by saying facts, listing data, emphasizing authority, etc. (Wei et al., 2016). The presentation of content needs to meet the user‘s thinking and logic, which overlaps with ‘resorting to logic’. However, because ‘resorting to credibility’ is related to ‘individual authority’, some scholars (Toto et al., 2020) summarize it as ‘resorting to personality’. For example, ‘resorting to return’ is a kind of commitment and return (Wei et al., 2016), but because it helps to arouse the audience‘s emotions, some scholars have summarized it as ‘resorting to emotion’ (Toto et al., 2020). Exaggeration is specifically used to exaggerate, brag, fabricate, etc.to influence user judgment (Wei et al., 2016; Gang and Qiansha, 2022), stimulate users ‘impulse buying emotions, which overlaps with ‘resorting to emotions’. For example, the ‘resort to rewards ‘proposed by Gang and Qiansha (2022) overlaps with the ‘resort to rewards’ (Wei et al., 2016), the promise and the ‘resort to emotions’ (Toto et al., 2020). Therefore, the existing rich and diverse communication styles need to be further simplified and unified.

### 2.3 Research on the language style of anchors

Na et al. (2020) classified communication styles into task-oriented and interaction-oriented styles. On the one hand, task-oriented communication styles emphasize cost and efficiency, with a high degree of goal orientation and purposefulness; while, on the other hand, interaction-oriented communication styles stress the social and personal aspects, and possibly neglect the task at hand. In the same vein, language style refers to the convergence of functional vocabulary use between communicating parties (Ireland et al., 2011). When exploring the influence of enhancing the communication styles’ similarity between anchors and users on the purchase intentions of consumers, Na et al. (2020) revealed that the similarity of anchor-user communication styles augments the attractiveness of the anchor, fosters the user’s perception of quasi-social interaction, and triggers the excited and active emotional state of users; thereby, leading to an immersive experience and ultimately, stimulating the user’s willingness to purchase. In the study of online health consultation dialog, Chih et al. (2020) found that when the patient‘s problem language style is abstract, the doctor can obtain higher service evaluation by using abstract language style. When the patient‘s problem language style is specific, the doctor can obtain higher service evaluation by using specific language style. Xing et al. (2022) found that doctors ‘use of abstract language style in online medical consultation is more likely to affect individual health anxiety through perceived uncertainty and information load. Si et al. (2022) divided the language styles of live broadcast spokespersons into online language and standard language styles. In particular, standard language refers to the standard Mandarin Chinese, which is a rigorous and standardized official language. Contrary to this, online language is a variant of standard language, which presents a creative, novel, and non-official language, often strongly associated with the Internet environment and Internet community. Moreover, Si et al. (2022) carried out an empirical analysis and concluded that there exists an interactive impact between the identity type of spokesperson and the language style; specifically, consumers are able to perceive a higher matching between officials and standard language, as well as a higher matching between Internet celebrities and online language, which results in higher purchase intention of consumers. Obviously, different types of anchors’ language styles exert diverse effects on the consumers’ purchase intention.

Pennebaker (2011) summarized that although the vocabulary of language style only accounts for 0.04% of the vocabulary of language content, the vocabulary of language style accounts for as much as 55% of the common vocabulary (Xinhua and Yaoqi, 2017). Anchors using different language styles will bring different live broadcast experiences to consumers, thus affecting consumers’ purchase intention, the existing literature on the impact of anchors’ language styles on consumers’ purchase intention is relatively small, the existing research mainly focuses on the anchor-user language style similarity (Na et al., 2020), the anchor identity-language style matching and other (Si et al., 2022) related to the research, but Is there any difference in the effects of anchors’ language styles on different products (practical and hedonic products)? Is there any difference in the effects of anchors’ language styles on different audience characteristics (independent self or interdependent self)? And how should anchors adjust their language styles to fit the preferences of live audiences? These questions remain to be addressed.

## 3 Research hypothesis

### 3.1 The influence of the language style of the anchor on consumers’ willingness to purchase

Under the development of the Internet, e-commerce live streaming represents a new social e-commerce model that warrants the anchor to introduce and display products in real time; thereby, triggering the intentions and willingness of consumers to purchase. Reportedly, Gang and Qiansha (2022) point out that the incorporation of different language styles by anchors may bring diverse viewing experiences to consumers; thus, inhibiting or stimulating their willingness to purchase. This directly leads to significant changes in the sales volume of key products. Parallel to this, Aristotle’s rhetoric school entails 3 successful persuasion modes, namely: logical appeal, emotional appeal, and personality appeal (Gang and Qiansha, 2022). These modes are explained as persuading individuals with appropriate reasons, intimidating them, and moving persons with emotion. This view has been followed by many scholars (Wei et al., 2016; Toto et al., 2020; Gang and Qiansha, 2022). However, there are mainly the following problems: First, resorting to personality is a kind of credibility, arbitrariness, and authority, which requires anchors to have strong personal characteristics, and this kind of language style is mainly applicable to celebrities, netizens, entrepreneurs, and other individuals with a certain fan base and a certain degree of social influence, and is not applicable to ordinary anchors or vegetative anchors, and enterprises usually can only require or cultivate anchors to have basic Communication skills, such as appealing to logic and appealing to emotion, and it is difficult to require or cultivate the anchor “appealing to personality” language style; Secondly, the anchor in the daily live broadcasting process for the product features mainly appealing to logic language style, for the product experience mainly appealing to emotion language style. Due to the individualized differences of anchors (image, voice, tone, etc.), they will gradually form their own unique communication style (i.e., appealing to personality) during the process of explaining products and interacting with users, therefore, appealing to personality is not a separate and distinct type of communication, but is based on appealing to logic and appealing to emotion. To summarize. Combined with the basic theories of Aristotle’s rhetoric techniques, this paper observes multiple e-commerce live-streaming platforms and studios, this study categorizes the language styles of anchors in live shopping contexts into appealing to logical language styles and appealing to emotional language styles. The appeal to logic refers to the anchor to use cause and effect relationship and other logical language to promote the product, Truthful and well-organized information helps to persuade others, e.g., use of words such as “if-then,” “then,” “because,” etc. This sense of logic will eliminate consumers’ doubts about the authenticity of the product, thus influencing their willingness to buy and increasing product sales; resorting to emotion refers to the anchor to add emotional information in the persuasive message, Empathize with each other through the use of emotional words, such as “dear,” “family,” “homey,” “sisters,” “buddy,” “brothers” and other pronouns to enhance customer relations, also repeatedly using the phrases of “grab it quickly,” “no more” to stimulate the tension and excitement of the live audience (Yi et al., 2022), and increase the consumers’ willingness to buy. According to Hovland’s model of persuasion, the change of others’ attitudes consists of four basic elements: the persuader, the object of persuasion, the persuasive message and the persuasive situation, among which the persuader is the first element of persuasion. Blankenship and Craig (2011) believe that the language style is an important element of persuasion, whether it is by appealing to the logical style of language, or by appealing to the emotional style of language has a persuasive effect, on the one hand, the language style is the most important element of persuasion. Have persuasive effect, on the other hand, language style will make the listener process the information in a biased way, forming the support of persuasion; on the other hand, language style will affect the listener’s cognition of the existing concepts, so as to influence the listener’s attitude and judgment.

Therefore, this paper concludes that anchor language style positively affects consumers’ purchase intention and proposes the following hypotheses:

H1: The anchor’s language style exerts a positive effect on the consumers’ willingness to purchase.

H1a: The language style of the host that appeals to logic exhibits a positive influence on the consumers’ willingness to purchase.

H1b: The emotional language style of the anchorperson demonstrates a positive impact on the purchase intention of consumers.

### 3.2 The influence of the language style of the anchor on consumers’ perceived value

According to Sheth et al. (1991), the consumer’s perceived value can be classified into 5 main dimensions including product performance, consumer recognition value, consumer emotional value, and social and environmental value. Subsequently, Sweeney and Soutar (2001) further categorized the consumer’s perceived value into 4 key dimensions, namely: functional value (quality), functional value (price), emotional value, and social value. The language style of the anchor can be adopted as a persuasive strategy that impacts the subjective perceived value of consumers. Appeals to Logic language style specifically through the explanation of product parameters, introduce product features, emphasize the after-sales protection, explain the price concessions, etc., with facts, information, data and other logical language to eliminate consumer purchase concerns, thereby increasing the consumer’s perception of functional value, and ultimately enhance the consumer purchase Intention; Appeals to emotional language style specifically through the sharing of product experience, introduction of product use skills, introduction brand and product stories, care for consumers, help consumers solve problems and other ways to pull into the distance between the consumer, to enhance the emotions of both sides, thereby increasing the consumer’s emotional value perception, and ultimately enhance the consumer purchase Intention. Prominently Ting et al. (2022) suggest that the authenticity and charm of the live-streaming anchor’s language positively affect the emotional- and functional value of consumers. Furthermore, the emotional- and functional value exhibits a positive effect on the purchase intention of live-streaming marketing. Correspondingly, the authors expect that the language style of the anchor displays a positive influence on the consumer’s perceived value. Thus, the below 3 hypotheses are outlined in this study:

H2: The anchor’s language style exerts a positive effect on the perceived value of consumers.

H2a: The use of emotional language styles demonstrates a positive influence on the consumers’ perceived value.

H2b: The use of logical language styles has a positive impact on the perceived value of consumers.

### 3.3 Consumer perceived value has a positive impact on customer purchase intention

In the field of marketing, consumer-perceived value has attracted substantial attention and consideration. Notably, consumer perceived value refers to the subjective assessment of the benefits of a product or service by consumers after deducting the costs of experiencing the product/service; thereby, reflecting their specific comprehension of the product or service. Moreover, purchase intention is often employed as a key indicator to anticipate consumer behavior, in order to evaluate market efficiency. This indicates that the consumers exhibit a subjective and targeted willingness to purchase products/services. Further, perceived value is assumed an imperative prerequisite factor that impacts the consumers’ purchase of products or services (Chang and Wildt, 1994). Remarkably, Zeithaml (1988) demonstrated the psychological mechanism of consumers realizing value through services or products. Their study conclusions implied that consumers’ perceived value exhibits a positive effect on their purchase of products/services. Consistent with the past studies, this research article hypothesizes the following hypotheses:

H3: Consumer perceived value demonstrates a positive impact on the customers’ purchase intention.

### 3.4 The mediating role of consumer perceived value

The SOR theory emphasizes that external stimuli demonstrate a significant effect on the consumers’ perceived value, whereas consumers evaluate the extent of perceived value based on the external stimuli. Particularly, consumer perceived value serves as a significant factor that impacts their purchase behavior and willingness. The anchor triggers consumers’ perceived value of the product through real-time communication and interaction with consumers in the e-commerce live streaming mode. In the meantime, the language communication style of the anchor plays an imperative role in the formation and creation of consumers’ purchase intention. Therefore, the authors advocate that the consumer perceived value plays a mediating role between the anchor’s language style and consumers’ purchase willingness. Correspondingly, this study puts forward the following hypothesis:

H4: Consumer perceived value plays a mediating role between the anchor’s language style and the consumer’s purchase intention.

### 3.5 Product types regulate the relationship between the language style of the anchor and the perceived value of consumers

This research article divides product types into practical products and hedonic products, in order to further explore the persuasive effects of different anchors’ language styles on different products. In accordance with the previous studies, hedonic products commonly reflect emotional benefits. Therefore, consumers who are committed to seeking sensory and experiential pleasure are conveniently driven by emotions and purchase hedonic products (Botti and McGill, 2011). In particular, purchasing hedonic products can meet the consumers’ own sensory pleasure and emotional needs. Relatively, practical products typically reflect the functional role of the product, therefore consumers who are committed to seeking in-depth and instrumental goals are principally driven by cognition and purchase practical products. Apparently, consumers purchase practical products, in order to save money, have wide availability, and seek convenience and practicality (Botti and McGill, 2011). Aligned with previous studies, this paper represents the below hypotheses:

H5: There exists a correlation between product type regulation, the language style of anchors, and consumer perceived value.

H5a: In the purchasing context of practical products, the anchor’s language style that appeals to logic shall bring consumers a higher willingness to purchase, as compared to the language style of the anchor that appeals to emotion.

H5b: In the purchase context of hedonic products, the language style of the host that appeals to emotions shall bring consumers a higher willingness to purchase, as compared to the host’s language style that appeals to logic.

**Figure 1:**
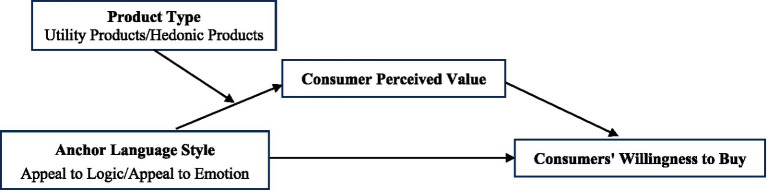
Research model diagram.

The theoretical model for this article is shown in Figure 1.

## 4 Study 1: hypothesis testing based on recall method

### 4.1 Research design

In order to ensure the validity and credibility of the research questionnaire, the estimation scale refers to domestic and foreign literature, and the topics are modified and supplemented in this study. Accordingly, the variables in the formal questionnaire include consumer purchase willingness (dependent variable), language style of the anchor (independent variable), product type (moderating variable), and consumer value perception (mediating variable). Meanwhile, all scales in this research study employed a 7-point Likert scale, where 1 represents “completely disagree” and 7 represents “completely agree.” The online questionnaire was distributed on several social platforms on the Internet. Consequently, a total of 236 questionnaires were returned by the respondents. Finally, 182 valid questionnaires were obtained after removing invalid questionnaires Table 1.

**Table 1:** Items and Sources of the Scale.

Variable name	Code	Measurement topics	Source
Product type	A1	Practical products refer to products with functional requirements, and higher emphasis is put on the prices, quality, functions, parameters, and other aspects.	Botti and McGill (2011)
	A2	Hedonistic products refer to products that satisfy emotional needs. Higher emphasis is placed on meaning, scenarios, and becoming a better version of ourselves. We pursue more value for money.	
Appeal to logic	B1	The anchor shall introduce the product’s material, parameters, and other indicators, and make appropriate comparisons with similar products.	Wei et al. (2016), Yi et al. (2022)
	B2	The anchor shall introduce the manufacturer and brand of the product, as well as the quality and after-sales guarantees.	
	B3	The anchor shall introduce the product’s preferential strength and constantly release benefits in the live broadcast room.	
	B4	The host shall introduce the price differences of the products on different platforms, illuminating the price benefits of the products in the live broadcast room.	
Appeal to emotion	C1	The anchor will conduct a product trial and visually present the product’s usage effect.	Wei et al. (2016), Yi et al. (2022)
	C2	The host shall conduct product usage instruction and teach skills and techniques related to the product.	
	C3	The anchor will talk about the experience of using the product and describe how to become a better person.	
	C4	The anchor will introduce the product and brand background story to help the viewers effectively understand the product and brand.	
Consumer perceived value	D1	When watching e-commerce live broadcasts, I enjoy relaxing and feeling happy.	Zeithaml (1988)
	D2	When watching e-commerce live broadcasts, I can learn some interesting or useful knowledge.	
	D3	I think the interaction of e-commerce live streaming is enjoyable.	
Consumer purchase intention	E1	I am willing to consider purchasing products during the live broadcast.	Na et al. (2020)
	E2	I am willing to continue watching the live broadcast and consider purchasing other products.	
	E3	I would recommend my friends and relatives to watch the live broadcast or purchase the products recommended by the live broadcast room.	

### 4.2 Reliability and validity testing

In this study, Cronbach α coefficient is used to test the reliability of the selected scale. In addition, SPSSAU software is utilized to carry out statistical estimations. The study results indicate that the Cronbach α coefficients of each variable range from 0.817 to 0.834, with a reliability coefficient value of 0.838, which is greater than 0.8. This reveals that the research data carries high reliability, therefore, it can be used for further analysis.

Subsequently, the validity was verified using KMO and Bartlett tests. The KMO value stood at 0.824, which was greater than 0.8, whereas the corresponding *p*-value of Bartlett’s test of Sphericity was reported to be 0.000. This reflects that the study data could be effectively extracted using Bartlett’s test of Sphericity. In addition to this, the 4 factors corresponded to the language style of the anchorperson; thereby, appealing to the logic and emotion, consumer’s perceived value, and consumer’s purchase intention, respectively. Parallel to this, the variance explanation rates of the 4 factors were documented to be 19.472, 17.406, 14.812, and 9.503%, respectively. Besides, the cumulative variance explanation rate after rotation was reportedly 61.193%, which was greater than 50%. This implies that the information content of the research items could be effectively extracted by the authors. Finally, there exists a corresponding correlation between the options and the factors when the absolute value of the factor load coefficient was greater than 0.4.

### 4.3 Correlation analysis

Prior to undertaking regression analysis, the authors need to perform correlation analysis on the variables used in the study, including the consumer perceived value, product type, consumer purchase intention, and language style of the anchor (appealing to logic and emotion) (Table 2).

**Table 2:** Correlation matrix of each variable.

	Average value	Standard deviation	Appeal to logic	Appeal to emotion	Consumer perceived value	Consumer purchase intention	Product type
Appeal to logic	5.485	0.869	1				
Appeal to emotion	5.254	0.947	0.409**	1			
Consumer perceived value	5.256	1.010	0.392**	0.527**	1		
Consumer purchase intention	5.288	0.879	0.274**	0.365**	0.548**	1	
Product type	1.154	0.362	−0.050	0.099	0.068	0.069	1

**Indicates *p* < 0.01.

### 4.4 Regression analysis

Using the language style of the anchor (appealing to emotion and appealing to logic) as the explanatory variable and consumer’s purchase intention as the explained variable, Model 1 is constructed in this study; subsequent to this, Model 2 is developed using the language style of the anchor (appealing to logic and appealing to emotion) as the explanatory variable and consumer’s perceived value as the explained variable; afterward, Model 3 is built using consumer perceived value as the explanatory variable and consumer’s purchase intention as the explained variable. The study findings indicate that the author’s language style (appealing to logic and appealing to emotion) exhibited a positive effect on the consumer’s purchase intention, therefore hypotheses H1, H1a, and H1b were established in this paper; further, the language style of the anchor (appealing to logic and appealing to emotion) demonstrated a positive influence on the consumer’s perceived value, thus, hypotheses H2, H2a, and H2b were proven in this study; finally, consumer’s perceived value exerted a positive impact on the consumer’s purchase intention. Hence, hypothesis H3 was also verified in this paper. At the same time, it is found that without considering the product type, the effect of appealing to emotional language style (*β* = 0.304, *p* < 0.001) on consumers’ purchase intention is greater than that of appealing to logical language style (*β* = 0.150, *p* = 0.048 < 0.05); the effect of appealing to emotional language style on consumers’ perceived value (*β* = 0.440, *p* < 0.001) is greater than that of appealing to logical language style (*β* = 0.212, *p* = 0.002 < 0.01), which may be due to the fact that with the development of the economy and the improvement of people’s living standards, consumers pay more attention to emotional interaction and emotional resonance than to logic and practical matters.

In terms of the mediation effect test, the anchor’s language style (appealing to logic and emotion) and the consumer’s perceived value were adopted as explanatory variables, whereas the consumer’s purchase intention was incorporated as the explained variable to develop Model 4. The comparison of regression results in Model 1 and Model 4 suggests that once a consumer’s perceived value is included as an independent variable, the significance of appealing to logic rose from Sig. = 0.048 < 0.05 to Sig. = 0.497 > 0.05, while the Sig. of appealing to emotion rose from Sig. = 0.000 < 0.05 to Sig. = 0.226 > 0.05. This reflects that the consumer’s perceived value plays a fully mediating role between the author’s language style and the consumer’s purchase intention; thus, enhancing the consumer’s purchase intention. As a result, hypothesis H4 is confirmed in this study, as depicted in Table 3.

**Table 3:** Regression analysis.

The model		Non-standardized coefficient		Standard Coefficient	*t.*	Significance
		*β*	Standard error	*B*		
Model 1 Y=Consumer purchase intention	(Constant)	2.975	0.434		6.860	0.000
	Appeal to logic	0.152	0.076	0.150	1.987	0.048
	Appeal to emotion	0.282	0.070	0.304	4.027	0.000
Model 2 Y=Perceived value of consumers	(Constant)	1.437	0.448		3.210	0.002
	Appeal to logic	0.247	0.079	0.212	3.133	0.002
	Appeal to emotion	0.469	0.072	0.440	6.492	0.000
Model 3 Y=Consumer purchase intention	(Constant)	2.779	0.290		9.573	0.000
	Consumer perceived value	0.477	0.054	0.548	8.796	0.000
Model 4 Y=Consumer purchase intention	(Constant)	2.373	0.403		5.885	0.000
	Appeal to logic	0.048	0.071	0.048	0.681	0.497
	Appeal to emotion	0.085	0.070	0.092	1.214	0.226
	Consumer perceived value	0.419	0.065	0.481	6.396	0.000
Model 5 Practical Group Y=Consumer perceived value	(Constant)	1.170	0.474		2.469	0.015
	Appeal to logic	0.303	0.090	0.260	3.385	0.001
	Appeal to emotion	0.458	0.087	0.406	5.276	0.000
Model 6 Hedonistic Group Y=Consumer perceived value	(Constant)	3.956	1.366		2.897	0.008
	Appeal to logic	−0.107	0.199	−0.095	−0.539	0.595
	Appeal to emotion	0.373	0.141	0.466	2.650	0.014

In order to further test the moderating influence of product type, this research study categorized the questionnaire into 2 main groups based on the product type, namely: practical goods and hedonic goods. Thereafter, a multiple linear regression model was established, with the following mathematical expression: in the practical goods’ group, consumer perceived value (Y) = A1 appeals to logic X1 + B1 appeals to emotion X2 + constant, which represents Model 5. In the hedonic goods group, consumer perceived value (Y) = A2 appeals to logic X1 + B2 appeals to emotion X2 + constant, which represents Model 6. The data from Model 5 illustrates that A1 = 0.303, B1 = 0.458, and A1 < B1. This confirms that as compared to the language style of the anchor who appeals to emotion, the language style of the anchor who appeals to logic does not bring higher purchase willingness to consumers in the purchase context of practical products, which infers that hypothesis H5a does not hold. This may be due to the following three reasons: first, in the definition of product type (practical and hedonic products), it is mainly up to the subjects to define the purchased goods as practical or hedonic products according to the definition of product type, but the same product may have both practical and hedonic attributes at the same time (Dhar and Wertenbroch, 2000), so there may be bias in the selection of product type by some subjects; second, with the economic development and the improvement of people’s living standards, consumers pay more attention to emotional interaction and emotional resonance (resorting to emotional language style) than logic and practical matters (resorting to logical language style). have a bias in the choice of product type; second, with the development of the economy and the improvement of people’s living standards, consumers pay more attention to emotional interaction and emotional resonance (appealing to the emotional language style) than to logic and practical matters (appealing to the logical language style); third, using the recall method for the measurements, people’s memories will be fuzzy over time, and their memories of the details of the events and situations will be gradually lost. Which may lead to memory bias. Afterward, the data from Model 6 depicts that A2 = −0.107, B2 = 0.373, and A2 < B2. This illustrates that in the purchase context of hedonic products, the language style of the anchor who appeals to emotion shall bring higher purchase willingness to consumers, as compared to the language style of the anchor who appeals to logic; thus, confirming hypothesis H5b.

## 5 Study 2: hypothesis testing based on experimental method

### 5.1 Research design

This experiment used a 2 (anchor language style: appeal to logic vs. appeal to emotion) × 2 (product type: practical product vs. hedonic product) between-group design, with the main purpose of verifying the moderating role of product type in the effect of anchor language style on consumers’ perceived value. Since the same product may have both practical and hedonic attributes (Dhar and Wertenbroch, 2000), for example, a cell phone is merely a practical product for communication for some consumers, while for some consumers it is a hedonic product symbolizing status and position, the authors chose cell phones as stimuli based on previous research (Dhar and Wertenbroch, 2000; Minxue et al., 2023), and through different purchasing purposes (a product for oneself vs. gifts for others) to manipulate cell phone attributes (practical goods vs. hedonic goods). The questionnaire was distributed through the online questionnaire platform “Questionnaire Star,” and a total of 115 subjects participated in this round of experiment.

The formal questionnaire consisted of four parts, the first of which guided subjects through stimulus materials into situations with different anchors’ language styles and product types. In the practical product scenario: “Suppose your own cell phone is old and difficult to use, and now you plan to buy a new cell phone for yourself, and you happen to see the cell phone brand XIA (in order to exclude the potential influence of the subjects’ personal preference for the brand, XIA is a fictitious brand name for this experiment) that you care about in the process of browsing the live broadcasts is selling the product in the live broadcast room.”, in the hedonic product scenario: “Suppose your boyfriend/girlfriend’s birthday is coming up, you want to prepare a surprise for each other and plan to buy a cell phone, you happen to see that the cell phone brand XIA that you pay attention to is selling the product in the live broadcasting room in the process of browsing the live broadcasting.” Subsequently, the subjects were shown the content of the product introduction by the anchor of the live broadcast, and different anchor language style groups saw different product introductions. In the resort logic language style group, consumers see the anchor’s product introduction: “XIA’s newest cell phone, equipped with the third generation of Snapdragon 8 s mobile platform, the comprehensive performance continues to lead the way, the new AI camera function, AI comprehensive arithmetic is powerful; with 6.36 gold size, 1.5 K super visual sense screen, extremely narrow visual four equal sides screen, quad-curved back shell, curvature rounded; With Leica Optics Summilux lens, with Shadowhunter 900 customized image sensor, comparable to a professional camera; today’s live broadcast to receive coupons, enjoy discounts, quickly order to buy it”; in the resorting to emotional language style group, consumers see the anchor’s product introduction: “XIA’s newest cell phone, which is equipped with the Third-generation Snapdragon 8 s based on generative AI, providing a virtual assistant-like function for your life; adopting 6.36 gold size, comfortable and proper hand feeling; 1.5 K resolution screen, bringing you a more vivid and realistic display effect; adopting an extremely narrow bezel design, realizing an excellent field of vision; adopting the light and shadow hunter 900 to take clear and sharp photos with vivid details and real texture; I’m using it myself now. Really great to use, whether you use or send your loved ones are full of surprises, hurry to order and buy it.” The product introduction copy in the experimental material was altered from Taobao live used data material to manipulate the language style by emphasizing the different values of the same phone.

The second part of the questionnaire measured the subjects’ evaluation of the product, including the consumer’s perceived value and the consumer’s willingness to buy, using the same scales as in Study 1, and the third part of the questionnaire included the manipulation of related variables. The third part of the questionnaire consisted of a manipulation test of the relevant variables and measurement of interferences, including measurement of subjects’ perceptions of language style and product type, where product type was measured by informing subjects of the definitions of practical and hedonic products, which emphasize the functionality and utility of the product, and hedonic products, which emphasize the emotion and feelings of the product, and then asking subjects to rate the products offered (1 = completely practical products, 7 = completely hedonic products), Language style type was measured using the same measure and subjects were asked to rate the anchor’s language style (1 = complete recourse to logical language style, 7 = complete recourse to emotional language style). In order to avoid the influence of subjects’ personal preferences for the products in the experimental materials on the results of the experiment, the attractiveness of the products was measured with the statement “According to the anchor’s description of the product in the live broadcast, how attractive is this product to you” (1 = not at all attractive, 7 = very attractive). The fourth section of the questionnaire measured the subjects’ personal demographic information, including gender, age, etc. Individual-related characteristic variables such as individual cultural context (individualistic and collectivistic culture), individual time orientation (future-oriented and present-oriented), self-efficacy (high self-efficacy and low self-efficacy), and family member composition (only child and non-only child) were also measured.

### 5.2 Data analysis

Manipulation test: First, a manipulation test of the experiment was conducted. The results showed that the manipulation of product type was successful, and there was a significant difference (*F* = 260.586, *p* < 0.001) between subjects’ perceptions of hedonic products (*M* = 5.25, M > 4, SE = 0.120, SD = 0.899) and utility products (*M* = 2.46, M < 4, SE = 0.124, SD = 0.953). The manipulation of anchor language style was successful, with a significant difference (*F* = 232.307, *p* < 0.001) between subjects’ perceptions of appealing to logical language style (*M* = 2.45, M < 4, SE = 0.141, SD = 1.030) and appealing to emotional language style (*M* = 5.29, M > 4, SE = 0.123, SD = 0.965).

Test of control variables: the results showed that product attractiveness control was successful and there was no significant difference (*F* = 2.718, *p* = 0.102) in the evaluation of product attractiveness between appealing to logical language style group (*M* = 4.64, SE = 0.127, SD = 0.922) and appealing to emotional language style group (M = 4.39, SE = 0.093, SD = 0.732). There was no significant difference (*F* = 0.077, *p* = 0.782) in the evaluation of product attractiveness between subjects in the utility product group (*M* = 4.53, SE = 0.114, SD = 0.878) and hedonic product group (*M* = 4.48, SE = 0.105, SD = 0.786).

Main effect analysis: this paper tests the differences between anchors’ language styles on consumers’ perceived value and consumers’ purchase intention. The results found that there is no significant effect on consumer perceived value (*F* = 1.573, *p* = 0.212) when anchors adopt appealing to logic language style (*M* = 5.22, SE = 0.132, SD = 0.961) or when they adopt appealing to emotion language style (*M* = 5.02, SE = 0.099, SD = 0.783); there is no significant effect on consumers’ purchase intention (*F* = 2.604, *p* = 0.06) when anchors adopt appealing to logic language style (*M* = 5.06, SE = 0.108, SD = 0.784) or adopt appealing to emotional language style (*M* = 4.73, SE = 0.088, SD = 0.690). In addition, this paper verified the difference of product type on consumers’ perceived value and consumers’ purchase intention. It was found that there is a significant effect of utility products (*M* = 4.94, SE = 0.117, SD = 0.902) or hedonic products (*M* = 5.29, SE = 0.108, SD = 0.807) on consumers’ perceived value (*F* = 4.896, *p* = 0.029 < 0.05), This may be due to the fact that utility products help consumers to obtain functional value, while hedonic products help consumers to obtain emotional value, and the difference in the type of value perception makes utility products and hedonic products have a significant effect on consumers’ perceived value. There is no significant effect of utility products (*M* = 4.81, SE = 0.095, SD = 0.733) or hedonic products (*M* = 4.95, SE = 0.102, SD = 0.767) did not have a significant effect on consumers’ purchase intention (*F* = 0.985, *p* = 0.323).

**Figure 2:**
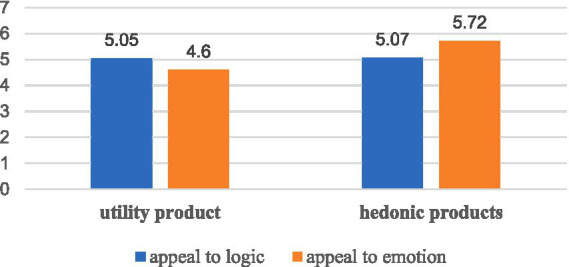
Interaction between product type and anchor language style.

Interaction effect of anchor language style and product type: in order to verify the effect of the interaction between anchor language style and product type on consumers’ purchase intention, this study takes consumers’ purchase intention as the dependent variable, and takes anchor language style and product type as the fixed factors, and uses the one-way F-test to test the interaction effect, and the results are shown in Figure 2. The results show that, for the utility product group, the anchor adopts the language style of appealing to logic (*M* = 5.05, SE = 0.161, SD = 0.850) has a more significant effect on consumers’ purchase intention (*F* = 5.890, *p* = 0.018 < 0.05) than the anchor adopts the language style of appealing to emotion (*M* = 4.60, SE = 0.097, SD = 0.540); for the hedonic products, the anchor’s use of a language style that appeals to emotion (*M* = 5.72, SE = 0.086, SD = 0.480) has a significantly higher effect on consumers’ purchase intention than (*F* = 16.503, *p* = 0.000 < 0.001) the anchor’s use of a language style that appeals to logic (*M* = 5.07, SE = 0.144, SD = 0.720).

### 5.3 Discussion of results

Through Study 2, it is found that there is no significant difference in the influence of different anchors’ language styles (appeal to logic and appeal to emotion) on consumers’ purchase intention, and that there is no significant influence of different product types (utility products and hedonic products) on consumers’ purchase intention. Then, we further compared the effects of two different anchors’ language styles on consumers’ purchase intention in different product types. It was found that the interaction between anchors’ language style and product type had a significant effect on consumers’ purchase intention. For utility products, the anchor’s use of logical language style is more likely to increase consumers’ purchase intention than the use of emotional language style; for hedonic products, the anchor’s use of emotional language style is more likely to increase consumers’ purchase intention than the use of logical language style. Therefore, the findings support hypothesis H5 (H5a and H5b). In order to further examine the effects of anchors’ language styles (appeal to logic and appeal to emotion) on consumers’ purchase intention in different cultural contexts (individualistic cultures and collectivistic cultures), different time orientations (future-oriented and present-oriented), different self-efficacy senses (high self-efficacy and low self-efficacy), different genders (males and females) and different family members’ compositions (one-child and non-one-child). In this study, we take consumers’ purchase intention as the dependent variable, and take anchors’ language style and cultural context, individual time orientation, self-efficacy, gender, and family member composition as fixed factors, and use one-way F-test to test the interaction effect, and the results are shown in Table 4. It is found that there is no interaction effect between anchor language style and cultural context (individualistic culture and collectivistic culture), individual time orientation (future-oriented and present-oriented), self-efficacy (high self-efficacy and low self-efficacy), gender (male and female), and family members (one-child and non-one-child), i.e., the effect of anchor language style on consumers’ purchasing intention will not be affected by the above individual factors.

**Table 4:** Influence of other factors.

Dependent variable	Individual factors	Fixed factors	Appeal to logic	Appeal to emotion	*p* value
Consumer purchase intention	Cultural contexts	Collectivistic cultures	*M* = 5.133	*M* = 5.278	*p* = 0.429 > 0.05
		Individualistic cultures	*M* = 5.026	*M* = 5.114	*p* = 0.645 > 0.05
	Time orientations	Present-oriented	*M* = 5.016	*M* = 5.017	*p* = 0.998 > 0.05
		Future-oriented	*M* = 5.194	*M* = 5.424	*p* = 0.279 > 0.05
	Self-efficacy	High self-efficacy	*M* = 5.219	*M* = 5.267	*p* = 0.768 > 0.05
		Low self-efficacy	*M* = 4.644	*M* = 4.970	*p* = 0.252 > 0.05
	Genders	Males	*M* = 5.333	*M* = 4.778	*p* = 0.196 > 0.05
		Females	*M* = 5.000	*M* = 5.226	*p* = 0.138 > 0.05
	Family members’ compositions	One-child	*M* = 5. 048	*M* = 5.156	*p* = 0.769 > 0.05
		Non-one-child	*M* = 5.058	*M* = 5.163	*p* = 0.514 > 0.05

## 6 Research conclusions and practical enlightenment

### 6.1 Research conclusions

In this study, the language style of the anchor is divided into two major types, namely: appealing to logic and appealing to emotion using empirical research, in order to examine the effect of the anchor’s language style in the e-commerce live broadcast environment on the consumers’ purchase willingness. The study results highlight that first, the language style of the anchor can be categorized into 2 main types: appealing to emotion and appealing to logic. Secondly, the language style of the anchor (appealing to emotion and appealing to logic) exerts a positive influence on the consumers’ purchase willingness through their perceived value. Thirdly, product type plays a partial moderating role in the correlation between the anchor’s language style (appealing to logic and appealing to emotion) and consumers’ perceived value. In specific, in the purchase context of hedonic products, the language style of the anchor appealing to emotion shall bring higher perceived value to consumers, as compared to the language style of the anchor appealing to logic; the language style of the anchor appealing to logic and appealing to emotion displays no significant effect on the consumers’ perceived value in the purchase context of utility products.

### 6.2 Practice enlightenment

In the e-commerce live-streaming scenario, the anchor should be customer-oriented and make proper use of the language styles of logic and emotional appeals based on the preferences of the key customer groups, in order to improve the user conversion rate in the live-streaming room. First, train anchors to master the skills related to appealing to logical language style and appealing to emotional language style. The platform can enhance the anchors’ business competency by extending script training for different language styles, effectively satisfying the needs of consumers, enhancing their perceived value, and bolstering their willingness to purchase. Secondly, cultivate the ability of anchors to be able to dynamically adjust their language style according to the real-time situation in the live broadcast room. The anchor is able to actively request real-time feedback from the customers through comments on the bullet screen, such as “Babies who want to quickly learn more practical information about products please reply ‘1’, “Babies who want to have a deeper understanding of more products and brand background stories please reply ‘2’, etc., in order to flexibly adjust the language style of the anchor. Alternative to this, the anchor can flexibly exhibit 2 different language styles based on the characteristics of the key customers’ age, gender, income, etc., while effectively interacting with the consumers. Third, the anchor should adopt different language styles for different product types. Specifically, for daily chemical products, household appliances, sanitary and cleaning products and other functional products are mainly used to appeal to logical language style, by emphasizing the product features and after-sales protection to eliminate user concerns, thereby enhancing live satisfaction and achieving consumer purchase intention; for gifts, luxury goods, theater performances, theme parks and other hedonic products are mainly used to appeal to emotional language style, through the brand story and consumer experience to achieve emotional resonance with the users, so as to enhance the satisfaction of the live broadcast and realize the consumers’ willingness to buy. Fourth, build a real-time feedback system for the anchor’s language style. On the one hand, the live room operation specialist should be based on the live room data and traffic, real-time judgment of the live room crowd portrait, and the dynamic portrait to the anchor for feedback, the live room anchor to adjust the live language style; on the other hand, the live room operation specialist according to the live room user length of stay, the live room shopping cart click rate, order rate, conversion rate and other indicators require the anchor to adjust the language style, in order to improve the effect of the live room On the other hand, enterprises can cooperate with third-party platforms and software companies to jointly develop a real-time evaluation system for the anchor’s language style, so as to help the anchor to enhance the effect of live broadcasting through different language styles in the live broadcasting process.

At this stage, language style is still subjective, personalized, difficult to imitate, difficult to quantify, difficult to measure and other characteristics, which undoubtedly brings difficulties to the management of the anchor’s language style in the live shopping scene. The era of artificial intelligence has arrived, the use of instrumentation to objectively monitor the speed of speech, intonation, timbre, meter and other elements of the anchor’s language style, and with the key indicators such as the length of stay of users in the live broadcast room, pop-up interactions, sales data and other key indicators, to form a set of scientific and objective assessment of the effect of the anchor’s language style is the direction of future research.

## Data availability statement

The original contributions presented in the study are included in the article/supplementary material, further inquiries can be directed to the corresponding author.

## Ethics statement

The studies involving humans were approved by Putian University Ethics Committee. The studies were conducted in accordance with the local legislation and institutional requirements. The participants provided their written informed consent to participate in this study.

## Author contributions

ZL: Conceptualization, Formal analysis, Funding acquisition, Methodology, Project administration, Resources, Supervision, Validation, Visualization, Writing – original draft, Writing – review & editing. SZ: Data curation, Investigation, Methodology, Software, Writing – original draft.
